# *Lactobacillus casei* Abundance Is Associated with Profound Shifts in the Infant Gut Microbiome

**DOI:** 10.1371/journal.pone.0008745

**Published:** 2010-01-18

**Authors:** Michael J. Cox, Yvonne J. Huang, Kei E. Fujimura, Jane T. Liu, Michelle McKean, Homer A. Boushey, Mark R. Segal, Eoin L. Brodie, Michael D. Cabana, Susan V. Lynch

**Affiliations:** 1 Division of Gastroenterology, University of California San Francisco, San Francisco, California, United States of America; 2 Department of Medicine, University of California San Francisco, San Francisco, California, United States of America; 3 Department of Pediatrics, University of California San Francisco, San Francisco, California, United States of America; 4 Department of Epidemiology and Biostatistics, University of California San Francisco, San Francisco, California, United States of America; 5 Ecology Department, Earth Sciences Division, Lawrence Berkeley National Laboratory, Berkeley, California, United States of America; University of Oxford, United Kingdom

## Abstract

Colonization of the infant gut by microorganisms over the first year of life is crucial for development of a balanced immune response. Early alterations in the gastrointestinal microbiota of neonates has been linked with subsequent development of asthma and atopy in older children. Here we describe high-resolution culture-independent analysis of stool samples from 6-month old infants fed daily supplements of *Lactobacillus casei* subsp. Rhamnosus (LGG) or placebo in a double-blind, randomized Trial of Infant Probiotic Supplementation (TIPS). Bacterial community composition was examined using a high-density microarray, the 16S rRNA PhyloChip, and the microbial assemblages of infants with either high or low LGG abundance were compared. Communities with high abundance of LGG exhibited promotion of phylogenetically clustered taxa including a number of other known probiotic species, and were significantly more even in their distribution of community members. Ecologically, these aspects are characteristic of communities that are more resistant to perturbation and outgrowth of pathogens. PhyloChip analysis also permitted identification of taxa negatively correlated with LGG abundance that have previously been associated with atopy, as well as those positively correlated that may prove useful alternative targets for investigation as alternative probiotic species. From these findings we hypothesize that a key mechanism for the protective effect of LGG supplementation on subsequent development of allergic disease is through promotion of a stable, even, and functionally redundant infant gastrointestinal community.

## Introduction

There is growing evidence that failure to develop a balanced immune response plays a key role in asthma and allergy development [1], [2], [3], and that environmental microbial exposure and host sampling of the developing gastrointestinal (GI) microbial community over the first year of life are crucial to immune response maturation [4], [5]. Culture-based approaches have suggested that the development of the GI microbiome is a progressive event beginning at birth and continuing until infants are weaned, with particular organisms acquired in distinct phases [6]. More recent, culture-independent studies have demonstrated that rather than a progressive colonization, the first year of life is characterized by fluctuating diversity of the microbial assemblage until convergence, with weaning, towards a GI community that more resembles that of an adult [7], [8], [9], [10]. As with adult GI bacterial consortia, inter-personal differences in GI microbial communities are evident in infants, particularly in the rate and stability of communities colonizing neonates [10], [11]. By 12 months old, the infant GI microbial community structure is relatively stable and the consortium largely resembles that of an adult, in which the Bacteroidetes and Firmicutes represent the two most dominant phyla [8], [9], [10].

A direct association has recently been demonstrated between the presence and abundance of specific microbial species in the GI tract of infants during the first 6 months of life and subsequent development of allergic disease at ages 1 and 2 [12], [13], demonstrating that early events in GI colonization precede development of allergic disease later in life. The first indication that a link existed between the GI microbiome and allergy was reported in the early 1980's in a study that described “dysbacteriosis” in infants with dermatological manifestations of food allergy, primarily due to low Bifidobacteria and Lactobacilli in combination with high numbers of species from the Enterobacteriaceae family [7]. Since then several studies have examined specific bacterial species in GI samples and demonstrated that their abundance correlated with atopy and asthma development [14], [15], [16], [17], [18]. A cross-sectional study of 1 year old infants in Estonia (low allergy prevalence) and Sweden (high allergy prevalence) demonstrated that more Estonian children possessed *Lactobacilli* and *Eubacteria* in their stool compared to Swedish children who were more likely to be colonized by *Clostridium difficile* [11]. A follow-up prospective study of stool samples from Estonian and Swedish children who were sampled over the first year of life and clinically followed up to 2 years of age, demonstrated that infants who developed allergy consistently exhibited lower levels of Bifidobacterial colonization compared to those that did not [16], [18]. Species-specific q-PCR analysis of the feces of 957 one-month-old infants in the KOALA birth cohort also demonstrated that a high abundance of *Escherichia coli* or *C. difficile* [12], [13] was associated with the development of eczema or atopy respectively [14].

The hygiene hypothesis suggests that a lack of microbial exposures during the crucial stages of immune maturation in infancy, results in immune modulation (Th2-biased response) that increases susceptibility to development of allergic disease [17]. Several studies have linked probiotic species with immunomodulation [14], [15], [16], [18], [19], [20], [21] and demonstrated their efficacy in protection against development of allergy and atopy. A randomized, controlled, double-blind study of 159 newborns, found that early feeding of *Lactobacillus casei* decreased the rate of atopic dermatitis at age two by 50% [22] and that this protective effect was sustained past infancy [23]. In animal models, probiotic supplementation has been shown to attenuate the respiratory inflammatory response [24] and reduce allergen-induced skin inflammation in sensitized mice [25]. Maassen and colleagues [26] demonstrated that of the eight *Lactobacillus* species used in their study of gut epithelial cytokine response, none induced local production of TGF-beta or IL-10, both of which are associated with a Th2 phenotype (a hallmark of atopic disease [27], [28], [29]). These observations are supported by other studies, which suggest that exposure to probiotics including *Lactobacillus* species, favors development of a Th1 phenotype and suppression of Th2 secreted cytokines [30].

The overall indication is that early events in gut microbial colonization play a key role in development of inflammatory disease at sites remote from the GI tract, and that a GI microbiome composed of beneficial bacterial species protects against allergy and atopic disease. Recent studies have demonstrated that the human gut is a densely populated, diverse bacterial microbiome [8], [9], [10], representing a complex host-microbial interaction. Therefore, it is likely that the species identified in previous studies may act as biomarkers for specific microbial consortia associated with, or protective against, allergic disease development. There is precedence for such a paradigm; several studies have demonstrated that bacterial community composition is dramatically altered in diseases such as obesity and periodontal disease [8], [31], [32] and that these changes are associated with altered consortium functionality [9].

While several studies have linked probiotics with improved clinical outcome, no study to date has examined the effect of supplementation on the overall GI microbiome to determine if the beneficial effects are due solely to high abundance of the species supplemented, or to a more global change in GI community structure. Here we describe culture-independent analysis of stool samples from 6-month-old infants at high risk for asthma development in the Trial of Infant Probiotic Supplementation (TIPS) study who were fed daily *Lactobacillus casei* subsp. rhamnosus (LGG) or placebo from birth to 6 months. Bacterial community composition was profiled using the 16S rRNA PhyloChip [33], [34], [35], a high-density, culture-independent microarray that can identify approximately 8,500 bacterial taxa (defined as groups of organisms that share at least 97% 16S rRNA sequence identity) in a single assay.

## Materials and Methods

### Ethics Statement

The Committee on Human Research at UCSF approved all study protocols, and all parents provided written, informed consent.

### Sample Collection

Stool samples from 6-month-old infants (n = 16) randomized in blocks of four to daily probiotic (*Lactobacillus casei* subsp. Rhamnosus (LGG; ATCC 53103; 1×10^9^ CFU) or placebo supplementation in the TIPS trial were used for this study. Samples were collected on the day prior to, or the day of the 6 month clinical visit from diapers using a scoop attached to the lid of a sterile collection vessel, prior to storage at 4°C and hand delivery or overnight mail to the study team. Samples were immediately banked at −80°C upon receipt.

### Sample Processing

Stool samples were thawed on ice prior to extraction of DNA using the UltraClean Fecal extraction kit according to the manufacturer's instructions (Mo Bio, CA). Universal primers 27F (5′-AGAGTTTGATCCTGGCTCAG-3′) and 1492R (5′-GGTTACCTTGTTACGACT T-3′; [36]) were used to amplify the 16S rRNA gene using 12 PCR reactions per sample performed across a gradient of annealing temperatures (48-58°C) to maximize diversity recovered. PCR reactions contained 0.02 U/µl Takara Ex Taq DNA Polymerase (Takara Bio Inc., Japan), 1× Takara buffer, 0.8 mM Takara dNTP mixture, 0.4 mg/ml bovine serum albumin (BSA) and 1.0 µM of each primer. PCR conditions were 1 cycle of 3 min at 95°C followed by 25 cycles of 95°C for 30 s, the gradient annealing temperature for 30 s, 72°C for 2 min and a final extension at 72°C for 10 min. A total of 100 ng of extracted DNA from the stool samples was used per PCR reaction. Amplified products from all 12 annealing temperatures were pooled, gel purified and processed for PhyloChip analysis as previously described [33], except that 500 ng of each amplicon was hybridized. Further details of the PhyloChip, its development and use are provided elsewhere [37], [38], [39], [40], [41].

### Microarray Analysis

Data sets were conservatively filtered, with taxa determined as present if ≥90% of probes in a probe set (for an individual taxon) were positive. Changes in probe-set fluorescence intensity are equivalent to changes in taxon relative abundance between samples. For taxa determined to be present in at least one sample, PhyloChip probe-set fluorescence intensity data was log transformed prior to analysis using packages in the R statistical environment [42]. Hierarchical cluster analysis (HCA) was performed on a Bray-Curtis dissimilarity matrix generated from PhyloChip fluorescence intensity data using the *vegan* package [43], followed by average linkage clustering.

A two-tailed Welch's T-test was used to identify taxa that were significantly altered in relative abundance in specific groups and adjusted for false discovery using the *qvalue* package as previously described. Significance was assigned with a p-value ≤0.05, q-value of 0.057. The 16S rRNA sequences of significant taxa were used to construct a neighbor-joining with nearest-neighbor interchange tree using FastTree [44] which was annotated using the Interactive Tree of Life (http://itol.embl.de/; [45]).

Correlation analysis of each individual taxon abundance against that of LGG (OTU_ID 3821) was performed using the *multtest* [46] package available as part of the Bioconductor suite of analysis programs [47]. Q-values were again generated for all p-values; taxa with r±0.5, p<0.05, q<0.15 were considered significant.

### Indices of Bacterial Community Phylogenetic Structure

Nearest-taxon index (NTI) and Net-relatedness index (NRI; [48], [49]) were calculated using the *picante* package [50]. A phylogenetic tree of representative sequences was constructed as described above and used together with taxon richness to calculate the mean phylogenetic distance (MPD) and mean nearest phylogenetic taxon distance (MNTD) using the phylogeny shuffle null model for each sample. MPD and MNTD values were used, as previously described [49], to calculate NRI and NTI values respectively for each sample. Inverse Simpson's diversity index [51] and Pielou's evenness [52] were calculated using the *vegan* package [43]. To accurately reflect community richness for this calculation, individual taxa were deemed to have an abundance of 0 if they did not meet the pf ≥0.9 criterion.

### Q-PCR Validation of LGG Presence and Relative Abundance

Quantitative PCR (Q-PCR) was performed to validate the presence and relative abundance of *L. casei* using primers based on PhyloChip probe sequences (LcF 5′-CGCATGGTTCT TGGCTGAAA-3′ and LcR 5′-ACAACAGTTA CTCTGCCGAC-3′). A total of 10 ng of DNA per reaction was used in triplicate, 25 µl Q-PCR reactions at an annealing temperature of 55°C. Regression analysis of inverse cycle threshold values plotted against array fluorescence intensity was used to confirm relative abundance of *L. casei* reported by the array and concordance between the two independent molecular methods.

## Results

### Bacterial Community Composition

**Figure 1:**
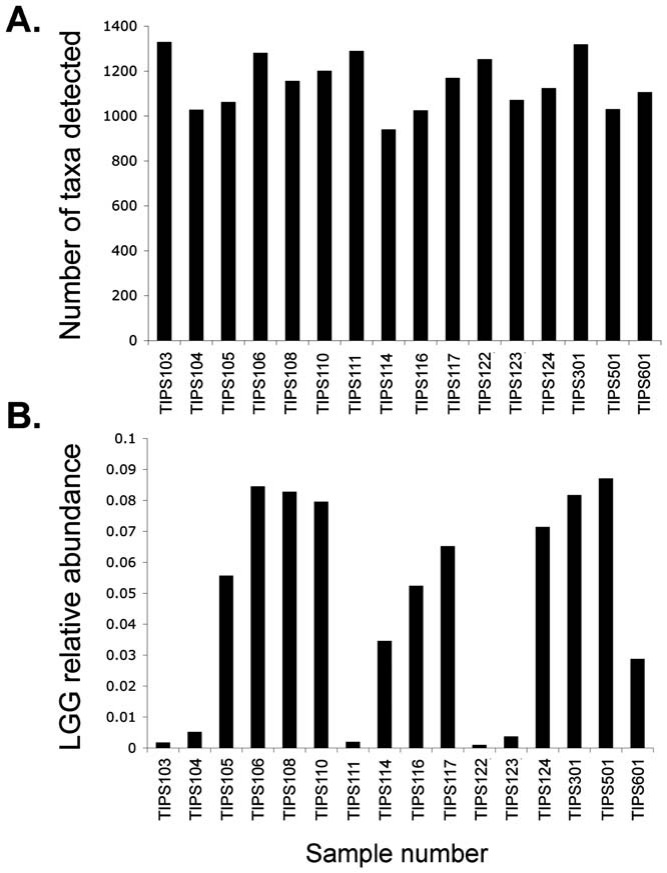
A. Community Richness. Bacterial community richness (number of taxa detected by 16S rRNA PhyloChip) in stool samples from 6 month old study subjects. **B.** **LGG Abundance**. LGG abundance (based on total fluorescence intensity) varies across subject samples.

In the original TIPS trial, infants were randomized in groups of 4, therefore the cohort investigated in this study consisted of subjects fed LGG and those fed placebo. However, to protect the integrity of the TIPS trial, investigators remained blinded to the nature of the daily supplement. A total of 1,988 taxa, representing 46 different bacterial phyla were detected across all samples (a complete list of taxa detected is provided in Supplementary material, Table S1). This represents considerably greater diversity than previously reported in a clone library study of healthy infant stool samples [10], likely due to the ability of the array to sample in parallel and detect species that represent as little as 0.01% of the bacterial community [34]. Community richness (number of taxa present) was broadly similar across all samples studied (mean number of taxa per sample was 1146±125; Fig. 1A). This suggests that none of these infants had received antibiotics, (characteristically associated with a rapid, dramatic decline in bacterial community richness [53], [54]), proximal to the date of sample collection. Community richness ranged from 950 (TIPS 114) to 1,333 taxa (TIPS 103). Inter-subject microbiota variation has previously been described in a study of infant GI microbiota [10], suggesting that daily probiotic supplementation with LGG (1×10^9^ CFU) did not result in dramatic domination of the communities by this species. Consortia were typically composed of members of the phyla Proteobacteria, Firmicutes, Actinobacteria and Bacteroidetes, which is in agreement with those detected in previous culture-independent studies of infant gastrointestinal microbiota [8], [9], [55]. At a more detailed phylogenetic level the greatest number of taxa detected belonged to the family Clostridiaceae, followed by the Enterobacteriaceae, Lachnospiraceae, Alteromonadaceae and Bacillaceae respectively.

Comparative analysis of LGG relative abundance in subject samples demonstrated that substantial differences existed across the cohort (Fig. 1B). To confirm differences in LGG abundance, independent Q-PCR analysis was performed on all samples with sufficient material (n = 11). Regression analysis demonstrated a significant correlation between the two independent molecular methods (r = 0.63; p<0.05), demonstrating concordance between the array and Q-PCR results and confirming the variation in relative abundance observed by the array in infant stool samples.

### Effect of LGG Abundance on Bacterial Community Structure

**Figure 2:**
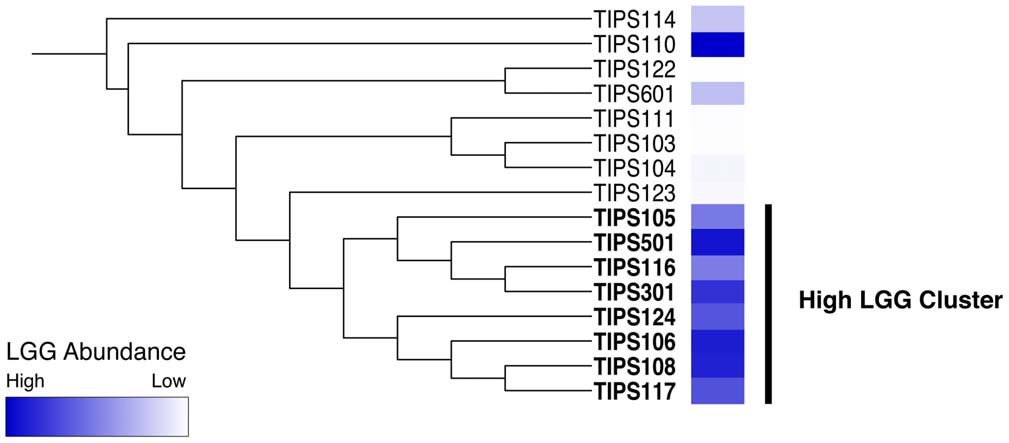
Hierarchical cluster analysis of infant stool samples. Hierarchical cluster analysis reveals that LGG abundance is associated with specific bacterial community structures.

Hierarchical cluster analysis was performed on all samples using a distance matrix representing differences in PhyloChip taxon intensities. This demonstrated the existence of a clear cluster which contained the majority of samples with high LGG relative abundance (Fig. 2), suggesting that the presence of LGG in high abundance was associated with a specific community composition. To identify the organisms that characterized those communities, all samples were ranked by abundance of LGG and the five samples with the highest abundance were compared to the five with the lowest abundance using a two-tailed Welch's t-test. Following adjustment for false discovery a total of 682 taxa demonstrated significant differences between the two groups, all of which were more abundant in the high LGG samples (a complete list of taxa exhibiting significantly different abundance is provided in Supplementary Table, S2). The fact that the relative abundance of a number of taxa was significantly higher in the high LGG samples indicates that changes in community structure associated with high abundance LGG are not simply due to a dilution effect, but putatively to promotion of other members of the GI microbiota. Taxa of interest that were significantly more abundant in the high LGG samples included, amongst others, nine related Lactobacillaceae (*L. crispatus* [two strains], *L. salivarius*, *L.sakei*, *L. manihotivorans*, *L. suntoryeus*, *L. kitasatonis*, *L. cypricasei* and *L. fuchuensis*), and a member of the Bifidobacteriaceae (*Bifidobacteriaceae* genomosp. C1).

**Figure 3:**
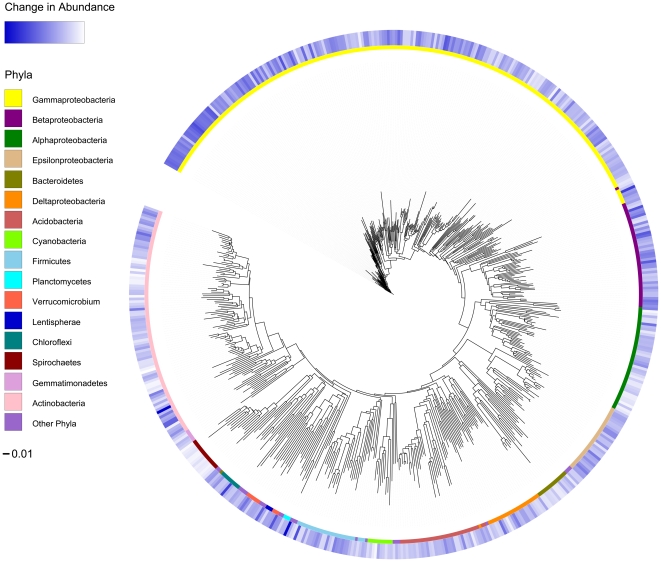
Significant Differences in Abundance of Taxa. Phylogenetic tree displaying taxa significantly increased in relative abundance in LGG dominated samples as a heatmap of fluorescence intensities in the outer ring. The inner ring displays the phylogenetic affiliation of each bacterial taxon at the level of class or higher. The scale bar indicates 0.01 nucleotide substitutions per base.

To determine if a high relative abundance of LGG impacted the abundance of phylogenetically related or, conversely phylogenetically distinct bacteria, community structure metrics Nearest Taxon Index (NTI) and Net Relatedness Index (NRI) were calculated (Table 1). Taxa promoted in the high LBB abundance communities had an NTI of 9.99 and NRI of 4.59, indicating that the species promoted in these communities were phylogenetically clustered at both the tips of the phylogenetic tree (NTI) and throughout the tree (NRI) relative to all bacteria detected. This clustering phenomenon is evident on a phylogenetic tree of all taxa that increased or decreased significantly in relative abundance; approximately 60% of the taxa that exhibited significantly higher abundance in the high abundance LGG communities belonged to the Proteobacteria and 37% of the total taxa were Gammaproteobacteria alone (Fig. 3). Various community metrics were also calculated for each individual sample and compared between high the LGG and low LGG samples by a two-tailed Welch's T-test (Table 1). Though the difference was small, Pielou's species evenness index was significantly higher in high LGG samples (p<0.041), demonstrating that communities with high abundance LGG were significantly more even than those with a low abundance of this species. Given the large size of the communities even this small, significant change in evenness is indicative of fundamental differences in the two communities. Species richness, community diversity (calculated using Inverse Simpson's index) Nearest Taxon Index (NTI) and the Net Relatedness Index (NRI) were not significantly different (p>0.05) between the two groups.

**Table 1:** Diversity and Phylogenetic Indices.

Sample	Taxon Richness	Pielou's Evenness	Inverse Simpson's Index	Nearest Taxon Index	Net Relatedness Index
*TIPS103*	*1333*	*0.9906*	*1185.09*	*−1.50*	*2.26*
*TIPS104*	*1014*	*0.9934*	*933.92*	*−1.04*	*−0.05*
TIPS105	1061	0.9932	974.79	1.25	1.39
**TIPS106**	**1285**	**0.9938**	**1187.24**	**−0.02**	**−5.30**
**TIPS108**	**1158**	**0.9942**	**1076.34**	**−0.30**	**−0.33**
**TIPS110**	**1203**	**0.9949**	**1129.03**	**−0.64**	**−0.25**
*TIPS111*	*1278*	*0.9938*	*1183.64*	*−2.40*	*−0.67*
TIPS114	950	0.9875	814.61	−2.77	−2.01
TIPS116	1032	0.9943	962.01	1.24	1.20
TIPS117	1161	0.9935	1069.16	−0.72	−2.31
*TIPS122*	*1253*	*0.9911*	*1124.22*	*−2.11*	*−2.56*
*TIPS123*	*1064*	*0.9926*	*970.82*	*0.16*	*1.94*
TIPS124	1117	0.9938	1033.53	0.82	−2.12
**TIPS301**	**1319**	**0.9939**	**1223.26**	**−1.74**	**−1.30**
**TIPS501**	**1020**	**0.9938**	**945.40**	**0.76**	**1.52**
TIPS601	1100	0.9932	1010.23	−2.22	−1.95

Samples in bold text were used as the examples of high LGG abundance and those in italics as examples of low LGG abundance in the t-test analysis.

### Correlation of LGG with Other Members of the Microbiota

We hypothesized that a proportion of the taxa that differentiated high LGG samples from other samples would be due to LGG-dependent interactions. Therefore we performed correlation analysis to identify the significant relationships that existed specifically between LGG and other taxa detected using all available samples. LGG was significantly correlated with 361 taxa (41% of which were also identified as significantly more abundant in high LGG samples; supplementary table S2). A total of 358 of these correlations were positive, 3 were negative. Positively correlated taxa included known probiotic species such as *Lactobacillus fuchuensis* and *Bifidobacterium bifidum* as well as several members of the Helicobacteraceae and a number of species that are known to produce antimicrobial compounds e.g. *Streptomyces coelicolor*. Interestingly *S. coelicolor* has been detected in the intestines of earthworms where it is particularly antagonistic to the anaerobic spore-forming bacteria [56]. Many of the positive relationships identified were with poorly characterized species, for example, the taxa most highly correlated with high LGG abundance were three Verrucomicrobia, two of which are unclassified and the third has the representative species *Prosthecobacter dejongeii* [57]. Characterized members of this recently described genus are fermenters and have been shown to encode a multitude of eukaryotic genes; their ancestors are hypothesized to have played an important role in the evolution of a proto-eukaryotic organism [58]. The three negative correlations revealed by this analysis were with *Bacteroides uniformis*, *B. merdae* and a swine intestinal clone classified as a member of the Lachnospiraceae.

## Discussion

The protective effect of specific probiotic species against diseases such as atopy (reviewed in [59]), irritable bowel syndrome [60], [61], [62] and neonatal necrotizing enterocolitis [63] amongst others has previously been demonstrated in a number of clinical studies. However, the species-specific mechanism of protection conferred by feeding commensal organisms remains elusive. We took advantage of an on-going clinical study, the Trial of Infant Probiotic Supplementation (TIPS), to pose the question whether probiotic supplementation results in a species-specific increase in relative abundance that accounts for protection or if there is a global effect on the complex GI microbial consortium. In the latter case, we hypothesized that comprehensive analysis of these communities would identify the consortia that act in concert to protect against allergic disease development.

PhyloChip analysis of stool collected from 6-month old infants who had received daily probiotic or placebo supplements permitted high-resolution profiling of the microbial assemblages present in these samples. Although we remained blinded to the identity of which infants received probiotic supplementation, comparative analysis of LGG relative abundance amongst the samples demonstrated clear differences in the abundance of this species. While we cannot be sure that all of these infants received probiotic supplementation, the fact that substantial differences in LGG abundance existed amongst our subjects allowed us to focus our analysis efforts on the effect of LGG abundance (high or low) on GI community composition. Though it is likely that those with the highest abundance received supplements and those with the lowest did not, it is difficult to determine whether samples TIPS114 and TIPS601 represent supplemented infants that have not retained the LGG as efficiently as other infants in the study, or whether they have received placebo but have higher than average abundance of LGG from other dietary sources. Cluster analysis of these samples, supported the hypothesis that LGG abundance was associated with a distinct community composition. The majority of samples with high LGG abundance clustered together and formed a group distinct from those with low LGG abundance.

Analysis of the phylogenetic differences characteristic of samples with high LGG revealed a large number of taxa increased in relative abundance in these communities. These included a number of known beneficial species belonging to the Lactobacillaceae and Bifidobacteriaceae in addition to species that, through their secondary metabolite production could conceivably influence GI consortium composition. Community phylogenetic metrics (NTI and NRI) demonstrated that the promoted taxa were strongly phylogenetically related. This suggests functional redundancy within GI communities that possess LGG in high abundance. Ecologically this attribute is characteristic of a stable, resilient consortia, resistant to sub-population overgrowth that can reduce host fitness [64]. In addition, samples with high LGG abundance were significantly more even; initial evenness of microbial communities has been suggested to preserve the functional stability of an ecosystem [65]. In the case of human hosts, overgrowth by pathogens, such as *Escherichia coli* in 1-month old neonates has previously been shown to be associated with a higher risk of developing eczema and this risk is increased with greater numbers of this species [12]. These observations point to a protective mechanism by which high LGG abundance results in promotion of other protective species in a community that is functionally redundant, more even, and possibly resistant to pathogen overgrowth.

Given these data, it is tempting to suggest that perturbation of the GI community, due to antibiotic administration or viral infection early in life, may affect microbial colonization patterns and permit outgrowth of specific resistant members of the community that influence immune development. Putatively, this provides an explanation for the relationships demonstrated between these factors and subsequent allergic disease development [66], [67]. Certainly there is support in the literature for such a model; several recent studies have demonstrated that host susceptibility to enteric pathogens is influenced by the GI bacterial community composition [68], [69] and Hrncir *et al* showed a link between gut microbiota, diet and development of T reg cells (key to maintaining immunological homeostasis) in germ-free mice [70]. Given the extent of GI community diversity and the inherent inter-personal variability in consortium composition, a comprehensive analysis of the pattern of colonization over the first year of life in well defined groups is necessary to address this, and many other questions fundamental to understanding the complexity of GI microbial factors that impact development of allergic disease.

In addition to a large number of commonly isolated or identified gut microorganisms, the PhyloChip also identified a range of taxa that are not commonly associated with this environment (supplementary table 1). There are a number of possible explanations for this, firstly the PhyloChip is a highly sensitive technique, rather than detecting the most abundant taxa present in a sample, it detects taxa in parallel to as little as 0.01% of the total abundance. Secondly, the representative organism for a given taxon (Table S1) may not have been reported previously in the gut environment, but other, related organisms in that taxonomic group may have been identified. Other culture-independent studies using high-throughput sequencing have also detected unexpected organisms in specific host niches; Eckburg *et al* [71] noted the presence of a novel, deeply branching lineage related to the Cyanobacteria by high-throughput sequencing in mucosal tissue and fecal samples while Andersson *et al* also identified a diverse stomach microbiota which included Cyanobacteria and Chlamydia sequences [72]. The more frequent use of high-coverage culture-independent approaches and repeated detection of these species in specific niches, validates their presence and suggests a role for these unusual organisms in human health.

Mucosal recognition and discrimination between commensal and pathogenic species is key to the regulation of immune homeostasis. This study certainly suggests that the relationship between LGG abundance and the immune response is significantly more complex than is inferred from studies of single species *in vitro*. Supplemental analysis to determine which taxa exhibited a relationship specifically with LGG abundance demonstrated that 41% of taxa differentiating high LGG from low LGG samples also exhibited significant correlations with LGG. Proposed mechanisms by which probiotic species are believed to elicit a beneficial effect include direct antimicrobial activity, competitive colonization, stimulation of immune responses and inhibition of virulence gene or protein expression by pathogenic species [73]. In addition to these possible factors, LGG also appears to elicit a profound effect on GI community composition. The beneficial effect of this species appears not to be due solely to its high abundance, but to the global changes in the bacterial community composition of the infant gut when it is present in high abundance. It is clear that a large number of other taxa detected in this study that have not previously been associated with, but are now potentially implicated in protection against allergic disease development, merit further study. To fully understand the functional implications of probiotic supplementation, parallel microbial consortium and host response gene expression analyses in clearly defined groups of probiotic or placebo supplemented infants are necessary.

The overall health effects of probiotic supplementation are strain-specific [59]. Nevertheless, there are potential general implications from this LGG-focused study which demonstrate that high abundance of this species is associated with a dramatic change in GI microbial community composition, impacting the relative abundance of a large number of taxa previously associated with either an increased or decreased risk for the development of allergy and atopy. Recent findings demonstrate that the GI microbiome is a unique, personalized assemblage at the species level [74]. Therefore there is potential for multiple components of this consortium to confer a protective effect. The data presented here suggests that promotion of a phylogenetically even, functionally redundant infant GI community, composed of a multitude of probiotic species, rather than a community dominated by a single beneficial species, may represent a key factor in protection against allergic disease development.

## Supporting Information

Table S1 Total taxa detected by PhyloChip. A list of all taxa detected by the 16S rRNA PhyloChip in all samples.
(0.38 MB XLS)

Table S2 Significantly correlated and T-tested taxa. A list of taxa that are significantly correlated, positively or negatively, with LGG together with those that demonstrate significant differences in abundance between high LGG (G1) and low LGG samples.
(0.25 MB XLS)
